# Non-valvular left atrial papillary fibroelastoma revealed by embolic stroke

**DOI:** 10.1093/ehjcr/ytag728

**Published:** 2026-09-28

**Authors:** Thibault-Marie Azevedo Mendes, Manuel Azevedo Mendes, Patrizio Lancellotti, Natzi Sakalihasan

**Affiliations:** Department of Cardiology, CHU de Liège, Avenue de l’Hôpital 1, Liège 4000, Belgium; Department of Cardiology, CHU de Liège, Avenue de l’Hôpital 1, Liège 4000, Belgium; Department of Cardiology, CHU de Liège, Avenue de l’Hôpital 1, Liège 4000, Belgium; Department of Cardiology, CHU de Liège, Avenue de l’Hôpital 1, Liège 4000, Belgium

## Summary

A 69-year-old woman presented with acute neurological deficits due to a cardioembolic pontine infarction. Multimodality cardiac imaging revealed a highly mobile, pedunculated mass arising from the lateral wall of the left atrium at the ostium of the left atrial appendage. Surgical excision was performed, and histopathology confirmed a papillary fibroelastoma. This case highlights the value of multimodality imaging in identifying a rare, non-valvular intracardiac source of embolism.

## Case description

A 69-year-old woman with an unremarkable history presented with abrupt right hemiparesis, hemianaesthesia, aphasia, and diplopia. Brain magnetic resonance imaging showed a subacute pontine infarction (*Figure 1A*, arrow), suggesting a cardioembolic mechanism.

**Figure 1:**
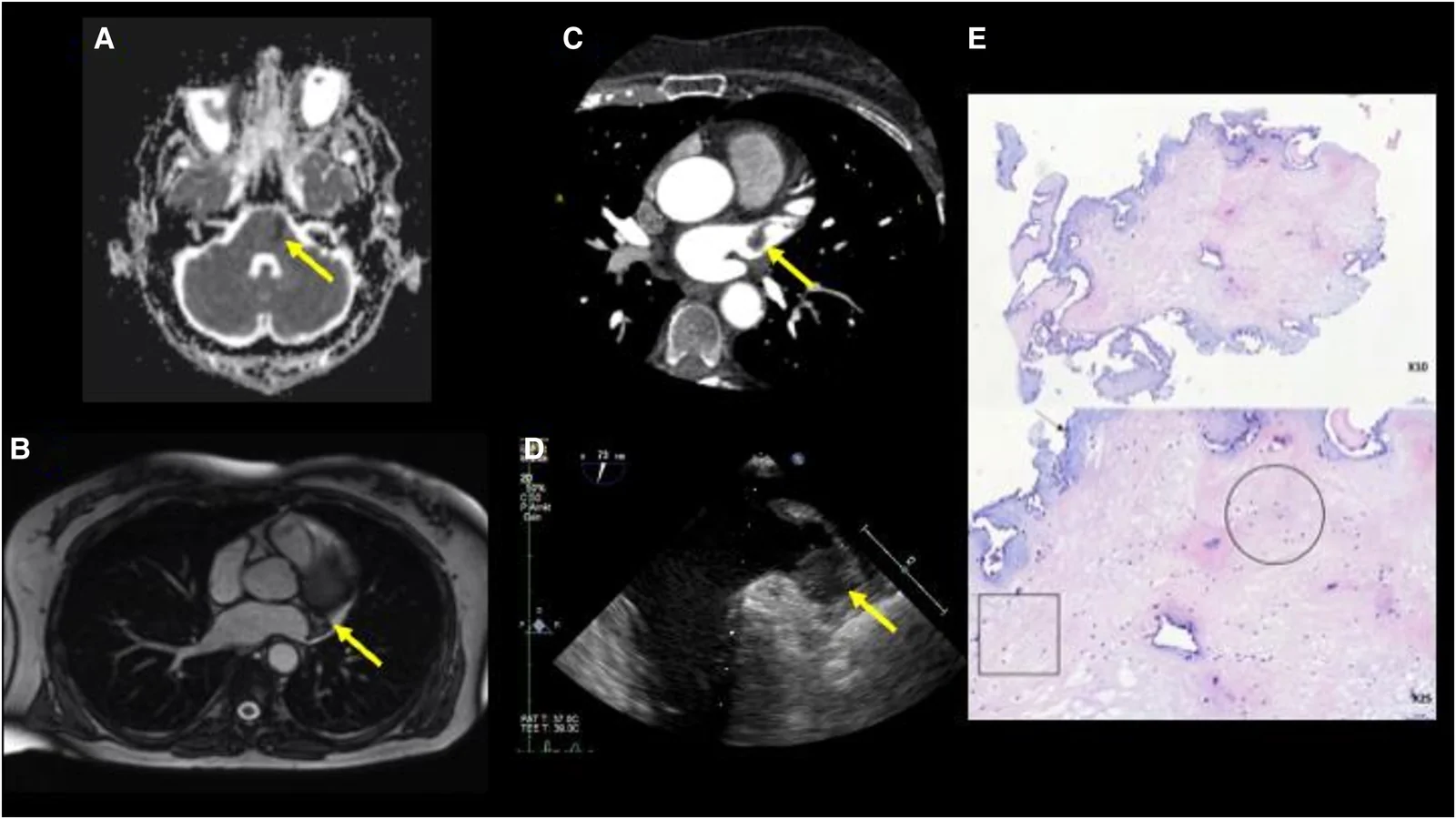
Multimodality imaging of a non-valvular left atrial papillary fibroelastoma presenting with embolic stroke. (*A*) Brain MRI showing a subacute pontine infarction (arrow). (*B*) Axial single-shot T2-TrueFISP cardiac MR image, on which the small mass is only faintly depicted (arrow). (*C*) Thin-slice (0.5 mm) cardiac CT clearly delineating the hypodense left atrial mass and excluding alternative embolic sources (arrow). (*D*) Transoesophageal echocardiography showing the highly mobile, pedunculated, multilobulated mass (arrow; see Supplementary video). (*E*) Histopathology (haematoxylin–eosin) confirming a papillary fibroelastoma.

On cardiac magnetic resonance (CMR) (*Figure 1B*, arrow), the lesion was only faintly depicted, on a single axial single-shot T2-TrueFISP image; the relatively thick cine slices (6–8 mm) did not clearly resolve this small mass. Cardiac computed tomography (CT) (*Figure 1C*, arrow), acquired with 0.5 mm slices, clearly delineated a small hypodense mass at the lateral wall of the left atrium at the ostium of the left atrial appendage and excluded alternative embolic sources. Transoesophageal echocardiography (*Figure 1D*, arrow, and Video) demonstrated a highly mobile, pedunculated, multilobulated mass at this site, oscillating within the atrial cavity and indicating high embolic potential (Supplementary material online, *Videos S1* and *S2*). The mass measured 12 × 7 × 2 mm.

Given the embolic presentation and marked mobility, surgical excision was performed 15 days after diagnosis. Macroscopically, the tumour had a friable, gelatinous ‘sea-anemone’ appearance; histopathology (*Figure 1E*) showed papillary fronds centred on a fibrous core and lined by a cuboidal-to-squamous epithelium, without inflammatory infiltrate or malignancy, confirming a papillary fibroelastoma.

More than 80% of papillary fibroelastomas arise from cardiac valves, most often the aortic and mitral valves^1,2^; a non-valvular left atrial origin is distinctly uncommon.^1^ Multimodality imaging is central to characterizing cardiac masses: small, highly mobile lesions may be under-resolved by standard CMR slice thicknesses and are often better depicted by high-resolution cardiac CT and transoesophageal echocardiography, as in this case.^3^ This complementary approach identified a rare non-valvular source of cardioembolism and guided prompt surgery.

The post-operative course was uneventful and, at nearly 10 years of follow-up, the patient had no recurrent neurovascular events and no neurological sequelae.

## Supplementary Material

ytag728_Supplementary_Data

## Data Availability

Data sharing is not applicable to this article as no new data were created or analysed in this study.
